# Standalone smartphone apps for mental health—a systematic review and meta-analysis

**DOI:** 10.1038/s41746-019-0188-8

**Published:** 2019-12-02

**Authors:** Kiona K. Weisel, Lukas M. Fuhrmann, Matthias Berking, Harald Baumeister, Pim Cuijpers, David D. Ebert

**Affiliations:** 1 Department of Clinical Psychology and Psychotherapy, Friedrich-Alexander University Erlangen-Nürnberg, Erlangen, Germany; 2 Department of Clinical Psychology and Psychotherapy, University of Ulm, Ulm, Germany; 3 Department of Clinical, Neuro and Developmental Psychology, Vrije Universiteit Amsterdam, Amsterdam, The Netherlands; 4 Amsterdam Public Health Research Institute, Amsterdam, The Netherlands

**Keywords:** Psychology, Outcomes research, Human behaviour

## Abstract

While smartphone usage is ubiquitous, and the app market for smartphone apps targeted at mental health is growing rapidly, the evidence of standalone apps for treating mental health symptoms is still unclear. This meta-analysis investigated the efficacy of standalone smartphone apps for mental health. A comprehensive literature search was conducted in February 2018 on randomized controlled trials investigating the effects of standalone apps for mental health in adults with heightened symptom severity, compared to a control group. A random-effects model was employed. When insufficient comparisons were available, data was presented in a narrative synthesis. Outcomes included assessments of mental health disorder symptom severity specifically targeted at by the app. In total, 5945 records were identified and 165 full-text articles were screened for inclusion by two independent researchers. Nineteen trials with 3681 participants were included in the analysis: depression (*k* = 6), anxiety (*k* = 4), substance use (*k* = 5), self-injurious thoughts and behaviors (*k* = 4), PTSD (*k* = 2), and sleep problems (*k* = 2). Effects on depression (Hedges’ *g* = 0.33, 95%CI 0.10–0.57, *P* = 0.005, NNT = 5.43, *I*^2^ = 59%) and on smoking behavior (*g* = 0.39, 95%CI 0.21–0.57, NNT = 4.59, *P* ≤ 0.001, *I*^2^ = 0%) were significant. No significant pooled effects were found for anxiety, suicidal ideation, self-injury, or alcohol use (*g* = −0.14 to 0.18). Effect sizes for single trials ranged from *g* = −0.05 to 0.14 for PTSD and *g* = 0.72 to 0.84 for insomnia. Although some trials showed potential of apps targeting mental health symptoms, using smartphone apps as standalone psychological interventions cannot be recommended based on the current level of evidence.

## Introduction

As mobile phone usage is ubiquitous, and mobile apps targeted at mental health are flooding the app market, mobile health (mHealth) interventions, which utilize mobile devices and technologies for mental health problems, are gaining popularity.^1,2^

Mental disorders are highly prevalent worldwide, can often have a detrimental impact on the life of affected individuals, and, to date, remain greatly undertreated.^3,4^ Although a wide array of evidence-based treatments exist, the majority of individuals with symptoms of mental disorders remain without treatment, even in high-income economies.^5,6^

Apart from structural treatment barriers such as availability, affordability, and time constraints, attitudinal factors play an even greater role in non-treatment-seeking behavior.^7^ Contributing factors hindering treatment uptake and treatment continuation include low perceived treatment need, poor mental health literacy, preference for self-reliance, and fear of stigmatization.^7,8^

Mobile devices could be utilized to overcome some of these issues. mHealth interventions utilizing apps on mobile devices have several benefits: (a) The threshold to use them is generally low and they provide the opportunity to engage individuals in need of treatment timely and anonymously by providing portable and flexible treatment; (b) mHealth might reach individuals who would otherwise not seek treatment;^9^ (c) most individuals already experience mobile devices to be an integral part of their everyday life and forthcoming generations are growing up as digital natives, where the use of apps for many different areas of life is becoming natural;^10^ (d) mHealth could be utilized to deliver large-scale interventions in emerging and low-income economies where resources for mental health are greatly limited,^4^ and (e) individuals can be supported in applying treatment-related skills in real life situations, in which behavior change is at its most vulnerable, and clinicians often struggle to support individuals appropriately.

The app-scape (app landscape) targeting mental health has increasingly been growing. According to a 2017 report, more than 318,000 health-related mobile apps were available for consumers of which 490 unique apps were targeted at mental health and behavioral disorders.^11^ A 2016 study scanning the app markets found 208 apps related to mental health or stress, most commonly targeting symptom relief (41%, 85/208) or general mental health education (18%, 37/208). The majority of identified apps did not mention any information on its effectiveness (59%, 123/208).^12^ Another study from 2018 rated the quality of depression apps available in German app stores with only 11% (4/38) showing some face validity, without any evidence on the effectiveness, and safety of any of the apps.^13^

Apps have been highly praised for their potential towards physical and mental health treatments.^14^ However, when considering using apps for mental health, there are also potential pitfalls. Disadvantages include technical aspects of delivery, such as screen size, battery life, system updates, technology requirements, as well as usage patterns, such as frequent but brief daily smartphone interactions, attentional competition between apps, short app lifespans, non-private settings, and data-security concerns.^15–17^

Unexpectedly, only very few studies have systematically examined the overall efficacy of mobile apps for mental health. A systematic review from 2013 by Donker et al., only found five apps, of which only three were evaluated in an RCT; targeting depression, anxiety and substance use, and with-in and between-group intention-to-treat effect sizes ranged from 0.29 to 2.28 at post-assessment and 0.01 to 0.48 at follow-up.^18^

Menon et al., conducted a systematic review on the feasibility of mobile phone apps and other mobile phone-based technology for psychotherapy in mental health disorders.^19^ Of 24 eligible articles, only eight involved smartphone apps. The eligible apps were found to be feasible and acceptable; however, no statistical analysis on the pooled efficacy was conducted.

There have also been previous systematic and meta-analytic disorder-specific examinations of efficacy, which found small effects for reductions in total anxiety scores from smartphone interventions compared to control conditions (*g* = 0.33, 95%CI 0.17–0.48, *P* < 0.01)^20^ and small effects for reductions in depressive symptoms from smartphone apps compared to control conditions (*g* = 0.38, 95%CI 0.24–0.52, *P* < 0.001).^21^ However, in both reviews, studies were included which did not primarily target depression or anxiety, including apps for memory training and attentional control, so that the effect of apps designed for anxiety and depression remains unclear, as well as the effect of apps individuals explicitly seek as treatment for a specific psychological disorder.

Although apps have been present for approximately ten years and are already being utilized by individuals seeking help for mental health problems, their overall efficacy remains unknown. Therefore, it is crucial to address this issue systematically. The aim of this meta-analysis is to investigate whether standalone psychological interventions for mental health delivered via smartphone apps are efficacious in reducing symptoms of mental disorders and self-injurious thoughts and behaviors (STBs) in adults with heightened symptom severity.

## Results

**Fig. 1:**
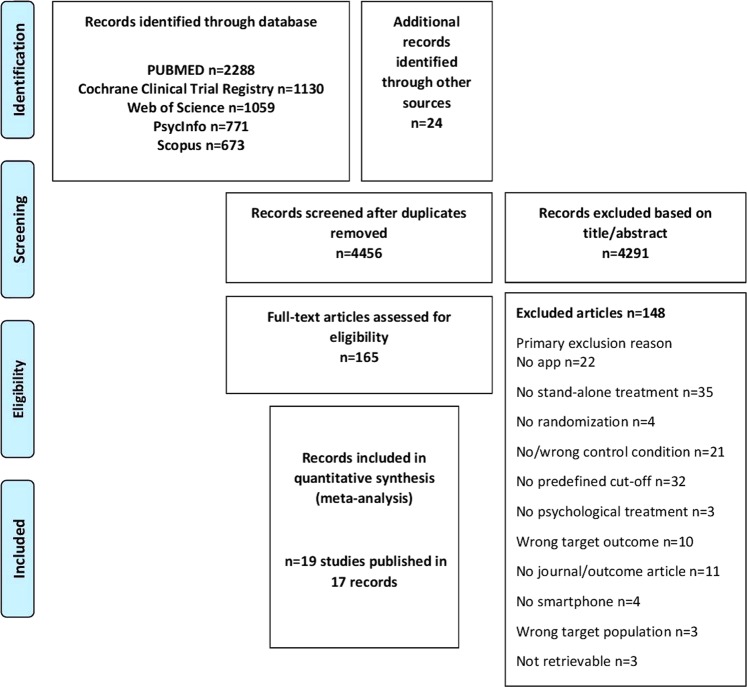
Study flow.

A total of 5921 records were retrieved from the electronic databases. After removal of duplicates and exclusion based on title and abstract, 165 records remained for full text screening. Seventeen publications describing 19 trials fulfilling all inclusion criteria between intervention and control groups remained and were included. Interrater reliability of full text eligibility check was good (*κ* = 0.64). Figure 1 displays the study flow (reference list of included trials Supplementary References).

All included studies were conducted in high-income countries (*k* = 11 USA, *k* = 3 Australia, *k* = 1 Israel, *k* = 1 the Netherlands, *k* = 1 South Korea, *k* = 1 Sweden, and *k* = 1 Switzerland). The primary study outcomes of the 19 included studies were depression (*k* = 6), anxiety (*k* = 4), posttraumatic stress disorder (*k* = 2), sleep problems (*k* = 2), substance use: smoking (*k* = 3), substance use: drinking (*k* = 3), and self-injury and suicidal behavior (*k* = 4). One study addressed smoking and drinking simultaneously. Several studies also assessed anxiety (*k* = 8) and depression (*k* = 12), either as primary or secondary outcomes.

All primary study outcomes only utilized self-report measures which were assessed at baseline and at post-assessment. Sample size of the included studies ranged from *n* = 34 to *n* = 983. The 19 included studies used different types of comparators: *k* = 2 no-treatment controls, *k* = 9 wait-list control groups, *k* = 3 information only, *k* = 4 sham group, and *k* = 1 daily mood chart.

All eligible apps were found to be based on some type of framework: cognitive behavioral therapy (*k* = 8), therapeutic evaluative conditioning (*k* = 1),^22^ attentional bias modification (*k* = 2), mindfulness (*k* = 1), behavioral modification (*k* = 1), theory of planned behavior (*k* = 1), breathing retraining (*k* = 1), monitoring with feedback (*k* = 1), decision aid (*k* = 1), problem-solving therapy (*k* = 1), and cognitive control (*k* = 1) (see Table 1).

**Table 1:** Characteristics of included studies.

Author year	Sample size included in review	Target outcome	Study inclusion	Participants Female: % (*N*)Age: *M* (SD)	Control group (active/inactive) Short description	Post-assessment period	Follow-up	Framework	Outcome
Arean 2016^53^	Total: 626 IG 1: 209 IG 2: 211 CG: 206	Depression	PHQ > 9, PHQ item 10 > 2	79% (494) 33.95 (11.84)	Inactive: Control app health tips	4-week	8-week, 12-week	IG 1: cognitive control IG 2: problem-solving therapy	Depression: PHQ-9
BinDhim 2018^54^	Total: 648 IG: 342 CG: 342	Smoking	Daily cigarette smokers	55% (340) 28.3 (10)	Inactive: Control app information	4-week	3-month, 6-month	Decision aid (psychoeducation, quit date)	Smoking: 30 day smoking abstinence
Birney 2016^55^	Total: 199 IG: 99 CG: 100	Depression	10 ≤ PHQ ≥ 19	76% (231)^a^ 40.65 (11.35)^a^	Inactive: Alternative care + WLC	6-week	10 weeks	CBT Mindfulness Positive psychology	Depression: PHQ-9
Clarke 2016^25^	Total: 36 IG: 18 CG: 18	Insomnia	PSQI ≥ 4	75% (18) 19.11 (2.51)	Inactive: Control app sham	8 days	No	Attention bias modification	Sleeping problems: PSQI & APSQ
Dar 2017^56^	Total: 40 IG: 20 CG: 20	Smoking	At least 5 cigarettes a day	22.5% (9) np	Inactive: WLC	4-week	No	Behavioral modification: monitoring & feedback	Smoking: Cigarettes per day
Enock 2014^57^	Total: 326 IG 1: 158 CG 1: 27 CG 2: 141	Social anxiety	SIAS ≥ 35^b^	47.8% (205) 34.8 (11.4)	Inactive: CG1 WLC CG2 sham	Mean of first 5 assessments	1-month & 2-month FU in IGs	Attention bias modification	Social anxiety: SIAS & LSAS Depression: DASS & PSWQ
Franklin 2016 Study 1^22^	Total: 114 IG: 59 CG: 55	Self-injury & suicidal ideation	Two or more episodes of self-cutting in past month	80.7% (92) 23.02 (5.47)	Inactive: Control app	4-week	No	Therapeutic evaluative conditioning	Self-Injurious Thoughts and Behaviors: Non-suicidal self-injury episodes & self-cutting episodes Suicidal ideation
Franklin 2016 Study 2^22^	Total: 131 IG: 62 CG: 69	Self-injury & suicidal ideation	Two or more episodes of self-cutting in past month	84.05% (110) 22.91 (4.99)	Inactive: Control app	4-week	No	Therapeutic evaluative conditioning	Self-Injurious Thoughts and Behaviors: Non-suicidal self-injury episodes & self-cutting episodes Suicidal ideation
Franklin 2016 Study 3^22^	Total: 163 IG: 78 CG: 85	Self-injury & suicidal ideation	At least one suicidal behavior within the past year	58.89% (96) 24.5 (6.61)	Inactive: Control app	4-week	No	Therapeutic evaluative conditioning	Self-Injurious Thoughts and Behaviors: Non-suicidal self-injury episodes & self-cutting episodes Suicidal ideation
Gajecki 2014^58^	Total: 983 IG 1: 341 IG 2: 153 CG: 489	Drinking	AUDIT ≥ 6 for women and ≥8 for men	51.7% (1001)^c^ 24.72 (4.81)^c^	Inactive: No-treatment control	7-week	No	IG 1: theory of planned behavior monitoring & feedback, IG 2: monitoring & feedback, simulation of drinking events	Substance use: alcohol binge occasions, frequency, quantity
Horsch 2017^26^	Total: 151 IG: 74 CG: 77	Insomnia	Diagnosis of insomnia & ISI ≥ 7	62.3% (94) 39.66 (13.44)	Inactive: WLC	7-week	3 months in IG	CBT	Sleeping problems: ISI & PSQI Depression: CES-D Anxiety: HADS
Hur 2018^59^	Total: 34 IG: 17 CG: 17	Depression	DSM-5 diagnosis for Other Specified Depressive Disorder	88.24% (30) 23.71 (3.26)	Inactive: Mood chart control group	3-week	No	CBT	Depression: BDI-II Anxiety: STAI-X2
Kuhn 2017^60^	Total: 120 IG: 62 CG: 58	Posttraumatic stress	PCL ≥ 35	69.17% (83) 39.28 (np)	Inactive: WLC	12-week	6 months in IG	CBT Symptom monitoring	PTSD: PCL-C Depression: PHQ-8
Miner 2016^24^	Total: 49 IG: 25 CG: 24	Posttraumatic stress	PCL ≥ 25	72% (40) 45.7 (13.9)	Inactive: WLC	4-week	2 months in IG	CBT Symptom monitoring	PTSD: PCL-C
Pham 2016^61^	Total: 63 IG: 31 CG: 32	Anxiety	ASI-3 > 15 & OASIS > 7 & GAD-7 > 5	50.8% (32) np	Inactive: WLC + information	4-week	No	Breathing retraining	Anxiety: GAD-7 & PDSS-SR
Roepke 2015^62^	Total: 283 IG 1: 93 IG 2: 97 CG: 93	Depression	CES-D > 15	69.9% (197) 40.15 (12.4)	Inactive: WLC	4-week	6-week	IG 1: CBT & Positive psychotherapy IG 2: CBT & acceptance-based therapy	Depression: CES-D Anxiety: GAD-7
Stolz 2018^63^	Total: 90 IG: 60 CG: 30	Social anxiety	SPS > 22 or SIAS > 33 and SKID diagnosis social anxiety	65.56% (59) 34.87 (np)	Inactive: WLC	12-week	6-month	CBT	Social anxiety: SPS & SIAS & LSAS Depression: BDI-II
Tighe 2017^64^	Total: 61 IG: 30 CG: 31	Suicidal ideation	PHQ-9 > 10, K10 > 24 & had suicidal thoughts in the previous 2 weeks	63.93% (39) 26.25 (8,13)	Inactive: WLC	6-week	12-week	Acceptance-based therapy	Suicidal ideation: DSI-SS Depression: PHQ-9
Witkiewitz 2014^65^	Total: 94 IG 1: 32 (IG 2: 33)^d^ CG: 29	Drinking & smoking	At least one episode of heavy drinking (5/4 drinks per occasion for men/women) in the past 2 weeks and reported concurrent smoking and drinking at least once a week	27.7% (26) 20.5 (1.7)	Inactive: No-treatment control	1-month	no	Mindfulness	Substance use: daily drinking & daily smoking

*np* not provided

^a^Based on total study sample size: *N* = 300

^b^Cut-off SIAS ≥ 35 applied in analysis, gender and age reported on total study sample (*N* = 429)

^c^Gender and age refer to complete study sample of *N* = 1932

^d^Not analyzed in this study

An overview of selected study characteristics can be found in Table 1. In total, 3681 participants were included in this study (overview of all trials and effects Supplementary Table 2).

### App and intervention components

In a further exploration of the studies, we reported the app and intervention components as described. All app features identified in the studies were used as a category to give an overview of how many other studies applied similar techniques. Of the 19 studies, eight (42%, 8/19) employed some type of symptom or behavior monitoring, active participant engagement was utilized in eight interventions which prompted participant engagement by explicitly requesting input (42%, 8/19), eight studies described mechanisms of tailoring based on context sensing, feedback or input (42%, 8/19), some sort of gamification was utilized in six studies (32%, 6/19), five studies sent intervention reminders based on adherence monitoring (26%, 5/19), four studies had some sort of social component added (21%, 4/19), two studies employed some sort of guidance (11%, 2/19), one study used a simulation of situations (5%, 1/19), and one study evaluated an intervention including a wearable device (5%, 1/19) (overview of app components is shown in Table 2).

**Table 2:** App & intervention components as reported in eligible studies.

Author year	Monitoring, e.g. EMA, symptom monitoring, progress monitoring	Participant engagement, e.g. participant input requested	Tailoring, e.g. context sensing, feedback or intervention content based on input	Gamification, e.g. comic format, upleveling	Adherence monitoring/reminders, e.g. text messages, emails, push notifications	Social component, e.g. forum use, enlist social support, social media use	Personalization, e.g. personal preferences, personal dashboard, use of photos, music, contacts	Guidance, e.g. phone calls, emails, text message, automated conversations	Simulation of situations	App linked to wearable device
Arean 2016^53^			✓	✓	✓					
BinDhim 2018^54^		✓			✓					
Birney 2016^55^	✓	✓	✓		✓					
Clarke 2016^25^										
Dar 2017^56^			✓							✓
Enock 2014^57^										
Franklin 2016 Study 1^22^				✓						
Franklin 2016 Study 2^22^				✓						
Franklin 2016 Study 3^22^				✓						
Gajecki 2014^58^	✓		✓						✓	
Horsch 2017^26^	✓	✓	✓		✓			✓		
Hur 2018^59^		✓	✓			✓				
Kuhn 2017^60^	✓	✓				✓	✓			
Miner 2016^24^	✓	✓				✓	✓			
Pham 2016^61^	✓	✓		✓						
Roepke 2015^62^				✓		✓				
Stolz 2018^63^								✓		
Tighe 2017^64^	✓		✓				✓			
Witkiewitz 2014^65^	✓	✓	✓		✓					
Sum	8	8	8	6	5	4	3	2	1	1

### Quality assessment

The initial interrater reliability between the two independent raters was considered good (*κ* = 0.67). Overall, risk of bias across trials was considerable. Thirteen (68%, 13/19) reported an adequate sequence generation, while only seven studies (37%, 7/19) reported on adequate allocation concealment. Seven studies (37%, 7/19) were rated as low risk concerning blinding of participants and personnel when participants received some type of sham treatment or app not knowing whether they received the treatment or not. All studies used self-report assessments so that blinding of outcome assessors was rated as not applicable. Twelve studies (63%, 12/19) took adequate measures to deal with missing data and described these methods at least partially. Concerning outcome reporting, only six (32%, 6/19) study registrations were retrievable in which studies had been registered and the outcomes were reported as originally stated. Although all trials were conducted in the last ten years and a priori trial registration has become standard practice, very few studies complied. Only one study (5%, 1/19) was assessed as having an overall low risk of bias (no bias detected) and three studies (16%, 3/19) were assessed with having a risk of bias in only one domain. More than half of the studies (53%, 10/19) were assessed as having some sort of bias in at least three domains (complete risk of bias rating is shown in Supplementary Table 1, Supplementary Figs 1 and 2).

### Depression

**Fig. 2:**
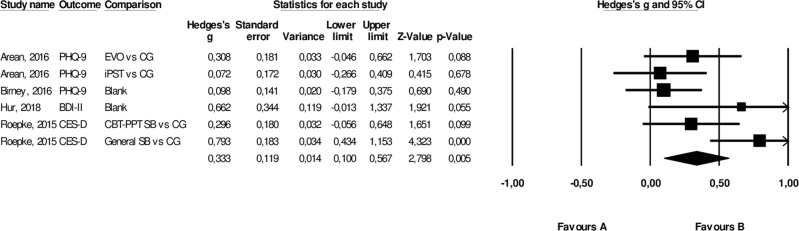
Forest plot pooled effect over target outcome depression.

Data on the primary target *depression* was pooled from six comparisons (*n* = 796). A significant pooled effect of smartphone apps compared to controls for *depression* was found with *g* = 0.33 (95%CI 0.10–0.57, *P* = 0.005). This translates to an NNT of 5.43. Heterogeneity between trials was moderate (*I*^2^ = 59%, 95%CI 0–83%). Figure 2 shows the forest plot.

Furthermore, *depression* was assessed in 12 comparisons (*n* = 1544) as primary or secondary outcome in apps for depression, anxiety, sleep problems, PTSD, and suicidal ideation. The pooled effect was significant in favor of smartphone apps compared to control groups with *g* = 0.34 (95%CI 0.18–0.49, *P* ≤ 0.001) which corresponds with an NNT of 5.26. Heterogeneity was moderate (*I*^2^ = 53%, 95%CI 10–76%) (forest plot Supplementary Fig. 4).

There was a significant effect of apps compared to controls for overall *depression* when analyzing trials that employed a wait-list control group (*k* = 8, *g* = 0.41, 95%CI 0.24–0.59, *P* ≤ 0.001, NNT = 4.39, *I*^2^ = 47%, 95%CI 0–76%) and a non-significant effect for other form of controls (*k* = 4, *g* = 0.17, 95%CI −0.00 to 0.42, *P* = 0.19, *I*^2^ = 36%, 95%CI 0–78%).

### Anxiety

*Anxiety* was investigated in four comparisons (*n* = 479). Differences between smartphone apps and controls were not significant (*g* = 0.30, 95%CI −0.1 to 0.7, *P* = 0.145) and heterogeneity was high (*I*^2^ = 75%, 95%CI 31–91%) (forest plot Supplementary Fig. 3).

*Anxiety* was also assessed in eight comparisons (*n* = 948) as either primary or secondary outcome of apps for anxiety, depression, and one app for sleep problems. In this analysis, we found smartphone apps to be superior to controls with a pooled effect of *g* = 0.43 (95%CI 0.19–0.66, *P* ≤ 0.001) which was significant, and relates to an NNT of 4.2. Heterogeneity was moderate to high (*I*^2^ = 66%, 95%CI 28–84%) (forest plot Supplementary Fig. 5).

Trials which employed a wait-list (*k* = 6) had a significant mean effect for overall anxiety of *g* = 0.49 (95%CI 0.27–0.71, *P* ≤ 0.001, NNT = 3.68, *I*^2^ = 47%, 95%CI 0–79%). Only two trials (*k* = 2) employed another type of control group which is why the effects were not pooled.

### Self-injurious thoughts and behaviors

*Suicidal ideation* was assessed in four comparisons (*n* = 286), of which three used the same intervention, the pooled effect was non-significant with an effect size of *g* = −0.14 (95%CI −0.37 to 0.1, *P* = 0.246). Heterogeneity was low (*I*^2^ = 0%, 95%CI 0–85%). *Self-injury* was assessed in three comparisons (*n* = 225) by the same authors evaluating the same intervention, and the pooled effect was not significant (*g* = −0.04, 95%CI −0.31 to 0.22, *P* = 0.746). Heterogeneity was low (*I*^2^ = 0%, 95%CI 0–80%) (forest plot Supplementary Figs 9 and 10).

### Substance use

Three comparisons (*n* = 780) investigated the effects on *smoking* and found a small significant effect of apps compared to control groups (*g* = 0.39, 95%CI 0.21–0.57, NNT = 4.59, *P* ≤ 0.001) and low heterogeneity between trials (*I*^2^ = 0%, 95%CI 0–93%).

Three comparisons (*n* = 1040) that investigated the effects on *drinking* did not find a significant effect of smartphone apps compared to controls (*g* = −0.03, 95%CI −0.22 to 0.17, *P* = 0.774, *I*^2^ = 48%, 95%CI 0–85%).

Effects on *substance use* were also pooled for *smoking* and *drinking* from five comparisons, with no significant difference between intervention and controls (*g* = 0.18, 95%CI −0.09 to 0.45, *P* = 0.2). Heterogeneity was high (*I*^2^ = 81%, 95%CI 54–92%) (forest plots Supplementary Figs 6–8).

### Posttraumatic stress disorder

Data on *PTSD* was not pooled over trials due to only two comparisons. Both trials evaluated the same intervention with both trials not finding significant effects in favor of the intervention versus control group (*g* = 0.14, 95%CI −0.22 to 0.5, *n* = 120, *P* = 0.439;^23^ and *g* = −0.05, 95%CI −0.6 to 0.51, *n* = 49, *P* = 0.873^24^).

### Sleep problems

Due to the limited amount of comparisons (*k* = 2) data for *sleep problems* was not pooled over trials. Both trials found significant effects in favor of the intervention group, with medium to large effects on *sleep problems* (*g* = 0.84, 95%CI 0.17–1.51, *P* = 0.014, *n* = 36, NNT = 2.23;^25^ and *g* = 0.72, 95%CI 0.39–1.05, *P* ≤ 0.001, *n* = 151, NNT = 2.56^26^).

## Discussion

Only 19 eligible trials were identified which evaluated the efficacy of a smartphone app designed to treat mental health symptoms in a randomized controlled trial, although hundreds of apps for mental health are available in the consumer app markets. This evaluation gap is in line with previous findings.^13,27^ All trials were conducted in high-income countries.

Significant pooled effects were found for depression (*g* = 0.33) and smoking (*g* = 0.39), with small effect sizes and moderate heterogeneity. No significant pooled effects were found for anxiety, alcohol use, and STBs. Additionally, when exploring effects on depression (*g* = 0.34) and anxiety (*g* = 0.43), regardless whether this was the primary aim of the intervention, pooled effects were significant, but heterogeneity was moderate to high. These effect sizes are in line with previous research investigating the efficacy of apps for depression and anxiety regardless of whether the app was aimed at depression or anxiety, or another mental health domain.^20,21^ Both trials targeting PTSD did not find significant effects of the evaluated interventions, whereas both trials on insomniac complaints did, with medium to large effect sizes. Overall, risk of bias was found to be considerate.

Interestingly, effects for most mental health domains were much smaller than what has been found in meta-analytic reviews for digital mental health interventions delivered using the internet (for an overview see e.g. ref. ^28^). For example, whereas effects on depression were small in the present analysis, the most recent meta-analyses on digital interventions targeting Major Depression found a mean standardized effect size of 0.90.^29^ Also, the non-significant effects on anxiety are somewhat surprising, given that meta-analytic reviews on internet-based interventions for anxiety found, when compared to passive control conditions, large effects; for example for social anxiety (*g* = 0.84)^30^ and general anxiety (*g* = 0.91)^5^ as well as for panic disorder (*g* = 0.83).^31^ The same goes for PTSD, for which the most recent review found an average pooled effect of 0.95,^32^ while the two only randomized trials that we were able to include in this study evaluating standalone apps for PTSD did not find significant effects. A different picture emerges when focusing on sleep problems. Although the effects of the two trials evaluating apps for insomniac symptoms were somewhat smaller than the average effects found for internet-based self-help interventions (*g* = 1.09), their confidence intervals overlap.^33^

These findings imply that the accumulating evidence for digital mental health interventions delivered through the internet as an effective mean to treat mental health disorders cannot be directly translated to digital interventions delivered via standalone mobile apps for all mental disorders.

Differences between findings in the present study for app-based standalone interventions targeting mental health symptoms and those often found for internet-delivered mental health interventions might be explained by systematic differences in patient, trial, or intervention characteristics. For example, guidance has been associated with higher effect sizes in digital mental health interventions,^34^ and many of the here included trials evaluated purely self-guided interventions without support from a professional. However, recent trials also indicate significant and moderate to large effects for unguided internet mental health interventions for some disorders such as anxiety or insomnia.^35,36^ Research has suggested that efficacy of apps is also dependent on long-term adherence to an app, otherwise the impact may be limited, and prompts where shown to increase effectiveness.^37^ Far fewer than half of the investigated studies employed such strategies; eight studies prompted participant engagement by explicitly requesting input and only five studies sent intervention reminders based on adherence.

Also, considering other app and intervention components utilized in the investigated apps, it seems that only a small portion of the uniquely available features of apps were actually used, which when applied correctly, might have the potential to compensate for other disadvantages of smartphone apps. It might also be helpful for individuals to have a fixed time and space to go through an intervention which might not be compatible with the general manner in which smartphones are used, e.g. when waiting in line or in a public space. The technological advantages of app-based interventions may not yet be so advanced to level out disadvantages of this approach; this might however change in the near future.

Overall, our knowledge of how to design effective mental health apps is very much at the beginning. Unlike established internet interventions, in which manuals for onsite psychotherapy can be directly translated and work very well, clinicians and researchers might need to start thinking outside of the box, and direct greater attention to the technological and persuasive design aspects of app-based interventions. Not being able to detect efficacy in many of the mental health domains might be based on an actual failure to deliver mental health treatment through mobile apps, however, this might also be based on the inefficacious manner in which the treatments were deployed and implemented.

Also, our findings do not refer to the use of apps as an adjunct to evidence-based treatments i.e. blended formats^38^ such as face-to-face^39^ or internet-based psychotherapy.^9^ One possibility to benefit from apps that already show small effects such as for depression, smoking, and sleep problems could be to have them integrated into a clinical setting in which a professional can monitor progress and provide additional support. This is a potentially relevant field which should be investigated systematically.

### Limitations

When interpreting the findings, the following limitations should be considered. First, heterogeneity was substantial in most analyses, as was risk of bias. As there were limited studies per disorder, also in the narrative description of study effects, findings should be interpreted with caution. Our findings of limited or non-existent efficacy of standalone treatment delivered by smartphone apps could also be due to the limited amount of studies available. Heterogeneity was not explored in a content-focused manner, nor did we investigate the effects of different features and components on efficacy or engagement. Overall, the limited efficacy does not mean that apps for mental health do not work in general but could also be an indication that the deployment and implementation so far has been unsuccessful. Also, we did not monitor or investigate whether the onboarding process happened in person or virtually which in turn might influence engagement. Engagement should be investigated in future studies.

Furthermore, the search was restricted to publications in English and German language. Due to the small amount of studies per disorder, the number of comparisons per disorder was limited, therefore not all study effects could be pooled, subgroup analyses were restricted, follow-up assessments were not examined, nor was publication bias explored. We originally planned to investigate (1) standalone smartphone apps for mental health and (2) hybrid apps and blended care apps. However, due to differing search strings and the already extensive scope of the primary research question, we decided to no longer investigate the second question within this manuscript.

### Future research

Although the potential smartphones bear to deliver mental health treatment is evident, future research needs to clarify whether the present limited efficacy holds true and identify in which circumstances their potential could be increased. The development of digital mental health services should be user-driven and solution-focused, including app developers, researchers and clinicians in the process.^37,40–42^ It is important to investigate app features and components, which make smartphone apps unique delivery modalities, such as context sensing, constant access and availability, high likelihood that prompts are received in daily life, and the combination possibilities with physiological assessments. The feature-driven approach could be helpful to understand working mechanisms of apps for mental health. Therefore, more specific aspects of mobile apps should be investigated and systematically tested, as well as long-term effects. Apps need to be implemented by utilizing persuasive design aspects and by exploiting full technological potential, which might improve effectiveness. It is also very likely that engagement is linked to efficacy of apps for mental health, which is why adherence, user profiles, and usage patterns should be further investigated.

### Conclusion

There remains a lack of generalizable evidence to support particular standalone smartphone apps for mental health as a substitute to conventional mental health treatment. Although we found a small effect for depression as well as indications for the use of apps for smoking and sleep problems, heterogeneity was substantial and overall risk of bias was high. Moreover, the insignificant findings on STBs, PTSD as well as alcohol use highlight the need for discussing the potential harm of currently available apps, which might keep users away from evidence-based interventions while bearing a substantial risk of being ineffective.

## Methods

The review was conducted in accordance with the Cochrane Handbook for Systematic Reviews of Interventions and reporting is based on PRISMA.^43,44^ This study was registered in the International Prospective Register of Systematic Reviews of the National Institute for Health Research (https://www.crd.york.ac.uk/prospero/) under the ID CRD42017076515.

### Eligibility criteria

We included (a) randomized controlled trials which (b) investigated the effects of a standalone psychological intervention for a specific mental health domain (c) delivered via a standalone smartphone app (d) aiming to reduce symptoms of a mental disorder or STBs (e) which the app was specifically targeted at. The trials must have included (f) an adult (≥18 years) population (g) with heightened symptom severity of a mental disorder according to DSM-IV or DSM-V^45,46^ or STBs (h) assessed by a diagnostic instrument or a predefined symptom cut-off (i) which was identical to the primary target of the study (j) compared to a control condition. Only (k) published peer-review articles (l) in English and German were considered. We decided to include STBs as they are pervasive among individuals with serious mental disorders.

A psychological intervention was defined as an intervention aimed at behavior change and reduction of mental disorder burden specifically designed and implemented with the intention to treat symptoms of mental disorders.

### Search strategy

A systematic literature search was completed (until 8 February 2018) in Pubmed, PsycInfo, SCOPUS, Web of Science, and Cochrane Central Register of Controlled Trials. Key search terms included a combination of three major themes: smartphone apps and mobile health, mental disorders, and randomized controlled trials (search string for Pubmed Supplementary Methods).

### Study selection

After duplicate removal, all titles and abstracts were screened for potential eligibility after which full text articles were assessed for eligibility. Study selection was performed by two independent researchers (K.K.W. and L.M.F.). Interrater reliability is reported for the initial agreement on full text eligibility where values of kappa are rated as fair (*κ* = 0.4–0.59), good (*κ* = 0.6–0.74), or excellent (*κ* > 0.75).^47^ Disagreement was resolved through discussion. If no consensus could be achieved, a third researcher was consulted (D.D.E.).

### Data extraction

The following data was extracted: (a) authors, year of publication, citation, (b) study design (sample size, study inclusion criterion, type of control group, target outcomes), (c) sample characteristics (age, gender), (d) treatment (theory basis, app and intervention components), and (e) data for calculation of effect sizes (outcome data preferably intention-to-treat (ITT) data, study dropout rate, handling of missing data). If data was not retrievable from publication, study authors were contacted for clarification.

### Quality assessment

Study quality was assessed independently by two researchers (K.K.W. and L.M.F.) based on the six basic criteria of the Cochrane Risk of Bias Assessment Tool: random sequence generation, allocation sequence concealment, blinding of participants and personnel, blinding of outcome assessors, incomplete outcome data, and selective reporting. Based on predefined definitions, studies were rated as “low”, “high”, or “unclear” risk of bias in each of these categories. When self-report measures were used to assess outcomes, blinding of outcome assessors were rated as not applicable (NA). When a study protocol or trial registration was identified and the primary outcome was reported as previously stated, selective outcome reporting was rated as low, if neither existed, selective reporting was rated as unclear. Studies which were rated as low on all available criteria were rated as overall low risk of bias. Interrater reliability of the risk of bias assessment is reported.

### Statistical analyses

Trials with the same primary target outcome were pooled to generate a mean effect size for each investigated outcome. If more than one measurement per outcome was assessed, the mean of the effect sizes was used to provide one effect size per study per outcome, to generate a mean effect size, only instruments were used related to the primary measure of the disorder. For each comparison between a smartphone app treatment and a control condition we calculated the effect size Hedges’ *g* (*g*), the 95% confidence interval (95%CI) and *P*-value (*P*) for each target outcome based on the post-assessment values indicating the difference between the two groups (intervention group versus control group) at post-assessment. For additional interpretability from a clinical perspective the number needed to treat (NNT) is reported for positive significant effects according to the method by Kraemer and Kupfer.^48^ In cases in which too few comparisons (*k* < 3) were available to pool data, data is presented in a narrative synthesis. Due to expected heterogeneity between trials, a random effects model was applied in all analyses. If trials were multi-armed, reporting two comparisons to one comparison, we divided the sample size to avoid inflating power.^43^

Heterogeneity between trials is expressed by *I*^2^ and its 95% confidence interval to express the percentage of total variance which can be explained. It can roughly be divided into three thresholds: low (25%), moderate (50%), or high (75%).^49^ The confidence interval was calculated by using the formula provided by Borenstein.^50^

In an additional analysis, the overall effects of all comparisons providing data on anxiety and depression after treatment with a smartphone app were each considered, no matter outcome status as primary or secondary. Potential sources of heterogeneity between trials were investigated by conducting subgroup analyses on different types of control groups. The subgroup analyses were conducted according to the mixed-effect model, in this model subgroups are pooled with the random-effects model while tests for significant differences between subgroups are conducted with the fixed-effects model.

Indications for publication bias were explored by investigating the funnel plot visually, and conducting Egger’s test.^51^ To obtain an estimation of the pooled effect when accounting for missing studies, the Duval and Tweedie trim-and-fill analysis was performed.^52^

All statistical analyses were calculated with the comprehensive meta-analysis (CMA) software, version 3 (Biostat, Inc.).

## Supplementary information

Supplement

## Data Availability

Data collected and used in this meta-analysis can be requested from the corresponding author.
