# Supplementary material for: Standalone smartphone apps for mental health—a systematic review and meta-analysis
Source: NPJ Digit Med. 2019 Dec 2;2:118. doi: 10.1038/s41746-019-0188-8 (PMC6889400; doi:10.1038/s41746-019-0188-8)
Supplement: Supplementary file 1 — Supplement [file 41746_2019_188_MOESM1_ESM.pdf]

Supplement:

Standalone Smartphone Apps for Mental Health – A Systematic Review and Meta-Analysis

Kiona K. Weisel[1], Lukas M. Fuhrmann[1], PhD Matthias Berking[1], PhD Harald Baumeister[2], PhD Pim Cuijpers[3], PhD David D. Ebert[1]

[1] Department of Clinical Psychology and Psychotherapy, Friedrich-Alexander University Erlangen-Nürnberg, Erlangen, Germany

[2] Department of Clinical Psychology and Psychotherapy, University of Ulm, Ulm, Germany

[3] Department of Clinical, Neuro and Developmental Psychology, Vrije Universiteit Amsterdam, The Netherlands; Amsterdam Public Health research institute, Amsterdam, The Netherlands

Corresponding author:

Kiona K. Weisel

Nägelsbachstraße 25a

91052 Erlangen

kiona.weisel@fau.de

Supplementary Figure 1: Risk of bias plot over all studies

Supplementary Figure 2: Risk of bias plot per study

Supplementary Figure 3: Forest plot of pooled mean effect anxiety

Supplementary Figure 4: Forest plot of pooled mean effect depression over all studies

Supplementary Figure 5: Forest plot of pooled mean effect anxiety over all studies

Supplementary Figure 6: Forest plot of pooled mean effect substance use: smoking

Supplementary Figure 7: Forest plot of pooled mean effect substance use: alcohol use

Supplementary Figure 8: Forest plot of pooled mean effect substance use

Supplementary Figure 9: Forest plot of pooled mean effect self-injury

Supplementary Figure 10: Forest plot of pooled mean effect suicidal ideation

Supplementary Table 1: Risk of bias overview

Supplementary Table 2: Outcome overview by study between-group post-assessment

Supplementary Methods: Search String Pubmed

Supplementary References: Overview of included studies

Supplementary Figure 1: Risk of bias plot over all studies

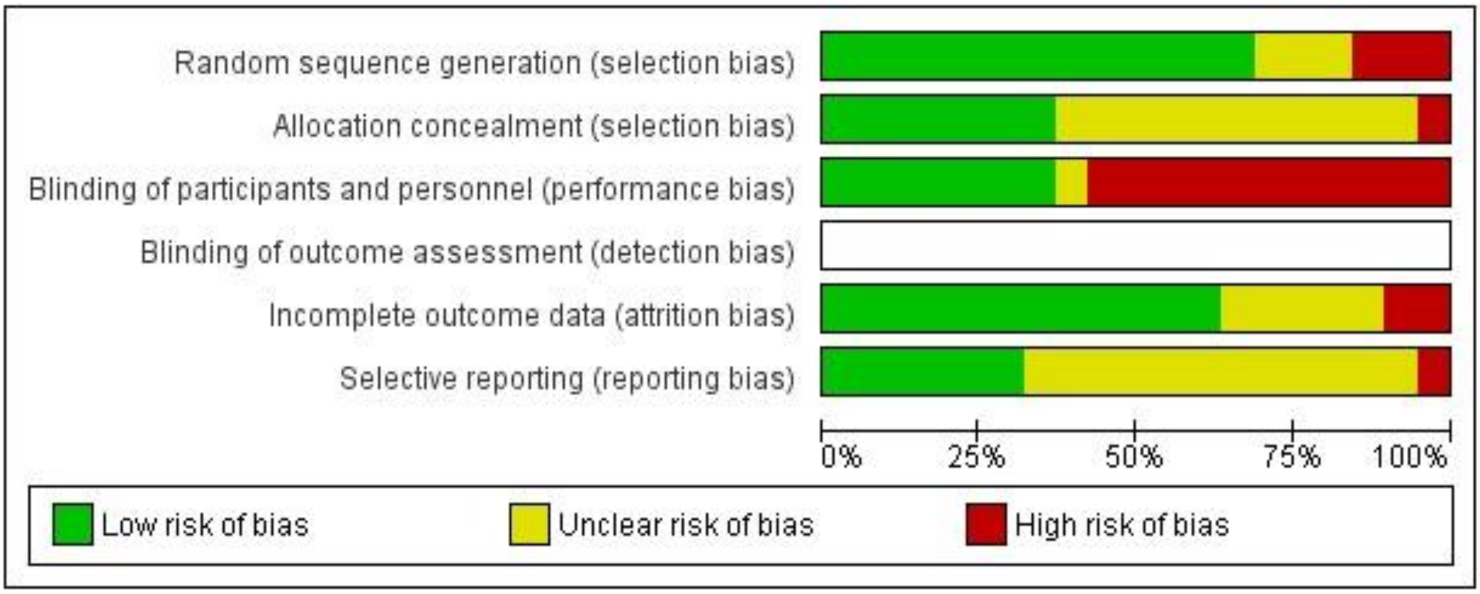

Risk of bias plot over all studies

Supplementary Figure 2: Risk of bias plot per study

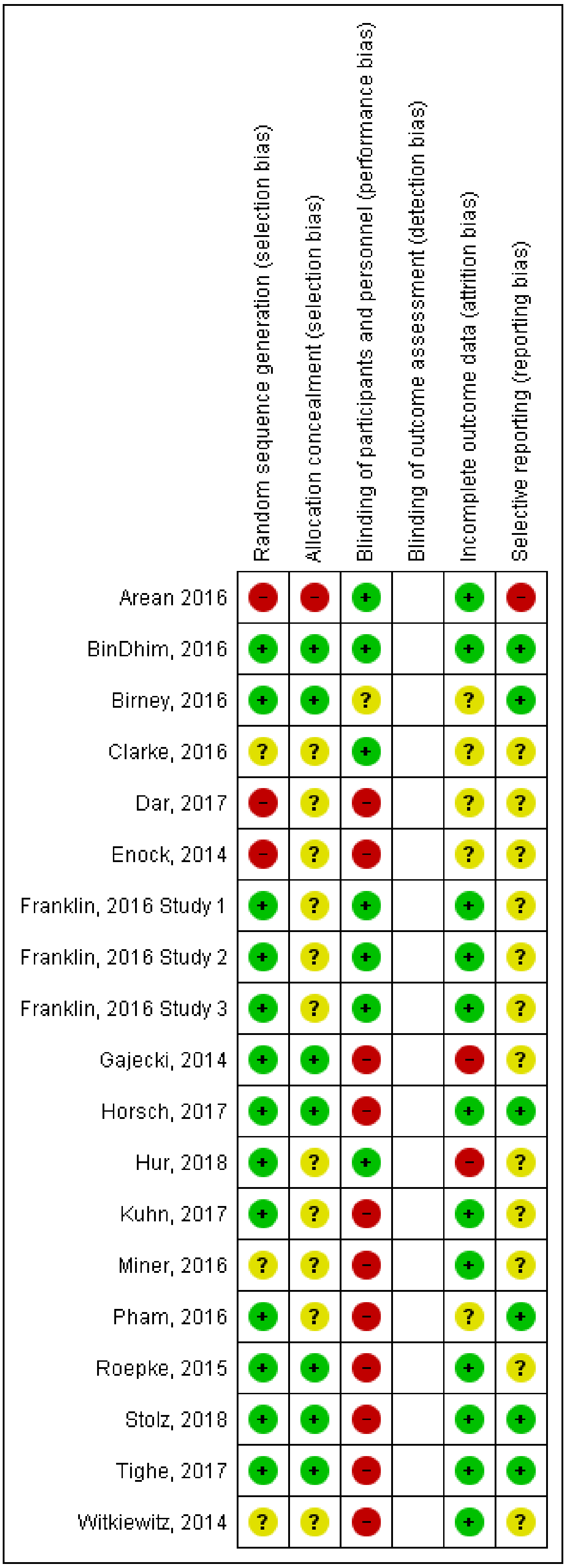

Risk of bias plot per study

Supplementary Figure 3: Forest plot of pooled mean effect anxiety

Study name	Outcome	Comparison	Hedges's g	Standard error	Variance	Lower limit	Upper limit	Z-Value	p-Value
Enock, 2014	Combined	CBM-A vs CG	0,645	0,226	0,051	0,202	1,088	2,856	0,004
Enock, 2014	Combined	CBM-A vs CON	-0,008	0,140	0,020	-0,283	0,266	-0,059	0,953
Pham, 2016	Combined	Blank	-0,084	0,249	0,062	-0,572	0,405	-0,335	0,737
Stolz, 2018	Combined	Blank	0,694	0,228	0,052	0,247	1,140	3,044	0,002
			0,300	0,206	0,042	-0,104	0,704	1,457	0,145

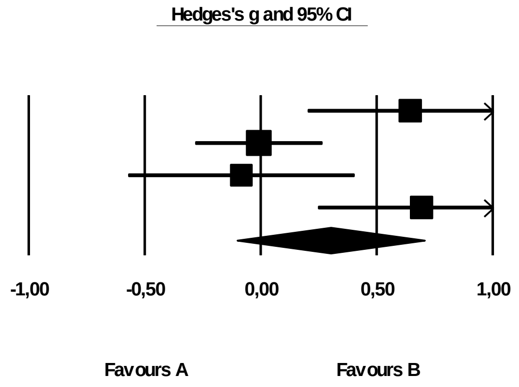

Forest plot of pooled mean effect anxiety

Supplementary Figure 4: Forest plot of pooled mean effect depression over all studies

Study name	Outcome	Comparison	Hedges's g	Standard error	Variance	Lower limit	Upper limit	Z-Value	p-Value
			Statistics for each study						
Arean, 2016	PHQ-9	EVO vs CG	0,308	0,181	0,033	-0,046	0,662	1,703	0,088
Arean, 2016	PHQ-9	iPST vs CG	0,072	0,172	0,030	-0,266	0,409	0,415	0,678
Birney, 2016	PHQ-9	Blank	0,098	0,141	0,020	-0,179	0,375	0,690	0,490
Enock, 2014	Combined	CBM-A vs CG	0,476	0,224	0,050	0,037	0,914	2,124	0,034
Enock, 2014	Combined	CBM-A vs CON	-0,025	0,140	0,020	-0,299	0,250	-0,176	0,860
Horsch, 2017	CES-D	Blank	0,571	0,165	0,027	0,247	0,895	3,457	0,001
Hur, 2018	BDI-II	Blank	0,662	0,344	0,119	-0,013	1,337	1,921	0,055
Kuhn, 2017	PHQ-8	Blank	0,228	0,182	0,033	-0,129	0,585	1,251	0,211
Roepke, 2015	CES-D	CBT-PPT SB vs CG	0,296	0,180	0,032	-0,056	0,648	1,651	0,099
Roepke, 2015	CES-D	General SB vs CG	0,793	0,183	0,034	0,434	1,153	4,323	0,000
Stolz, 2018	BDI-II	Blank	0,299	0,223	0,050	-0,138	0,735	1,341	0,180
Tighe, 2017	PHQ-9	Blank	0,707	0,261	0,068	0,195	1,218	2,709	0,007
			0,336	0,079	0,006	0,180	0,492	4,227	0,000

Hedges's g and 95% CI

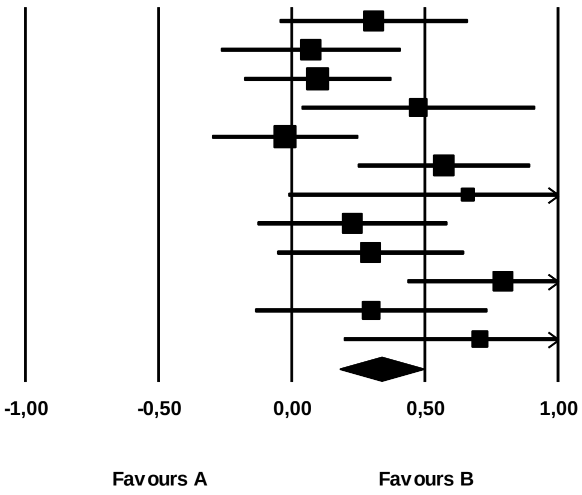

Forest plot of pooled mean effect depression over all studies

Supplementary Figure 5: Forest plot of pooled mean effect anxiety over all studies

Study name	Outcome	Comparison	Hedges's g	Standard error	Variance	Lower limit	Upper limit	Z-Value	p-Value
Enock, 2014	Combined	CBM-A vs CG	0,645	0,226	0,051	0,202	1,088	2,856	0,004
Enock, 2014	Combined	CBM-A vs CON	-0,008	0,140	0,020	-0,283	0,266	-0,059	0,953
Pham, 2016	Combined	Blank	-0,084	0,249	0,062	-0,572	0,405	-0,335	0,737
Stolz, 2018	Combined	Blank	0,694	0,228	0,052	0,247	1,140	3,044	0,002
			0,300	0,206	0,042	-0,104	0,704	1,457	0,145

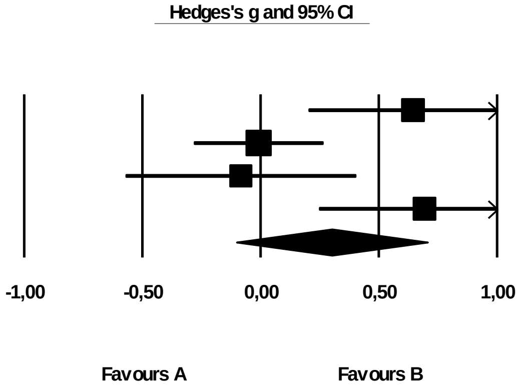

Forest plot of pooled mean effect anxiety over all studies

Supplementary Figure 6: Forest plot of pooled mean effect substance use: smoking

Study name	Outcome	Comparison	Hedges's g	Standard error	Variance	Lower limit	Upper limit	Z-Value	p-Value
			Statistics for each study						
BinDhim, 2018	abstinence	Blank	0,371	0,103	0,011	0,168	0,573	3,588	0,000
Dar, 2017	cigarettes per day	Blank	0,694	0,320	0,102	0,068	1,321	2,173	0,030
Witkiewitz, 2014	CSD smoking per smoking day	Blank	0,338	0,266	0,071	-0,183	0,860	1,271	0,204
			0,394	0,092	0,008	0,213	0,574	4,270	0,000

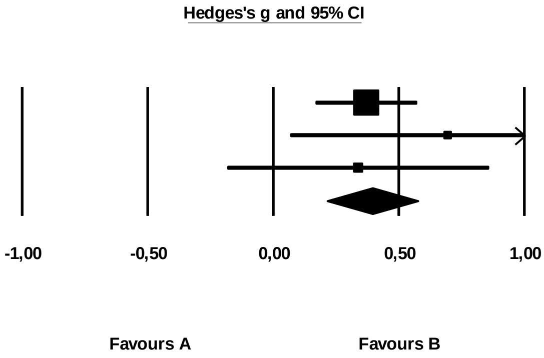

Forest plot of pooled mean effect substance use: smoking

Supplementary Figure 7: Forest plot of pooled mean effect substance use: alcohol use

Study name	Outcome	Comparison	Statistics for each study						
			Hedges's g	Standard error	Variance	Lower limit	Upper limit	Z-Value	p-Value
Gajecki, 2014	Combined	Party Planner vs CG	0,053	0,103	0,011	-0,149	0,255	0,515	0,607
Gajecki, 2014	Combined	Promillekoll bs CG	-0,161	0,084	0,007	-0,326	0,003	-1,923	0,054
Witkiewitz, 2014	Combined	Blank	0,227	0,265	0,070	-0,293	0,748	0,857	0,392
			-0,029	0,099	0,010	-0,223	0,166	-0,288	0,774

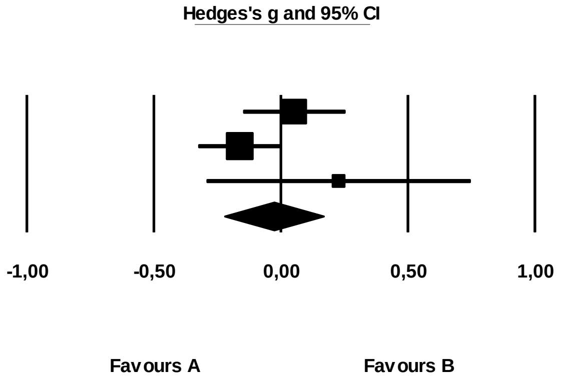

Forest plot of pooled mean effect substance use: alcohol use

Supplementary Figure 8: Forest plot of pooled mean effect substance use

Study name	Outcome	Comparison	Hedges's g	Standard error	Variance	Lower limit	Upper limit	Z-Value	p-Value
			Statistics for each study						
BinDhim, 2018	abstinence	Blank	0,371	0,103	0,011	0,168	0,573	3,588	0,000
Dar, 2017	cigarettes per day	Blank	0,694	0,320	0,102	0,068	1,321	2,173	0,030
Gajecki, 2014	Combined	Party Planner vs CG	0,053	0,103	0,011	-0,149	0,255	0,515	0,607
Gajecki, 2014	Combined	Promillekoll bs CG	-0,161	0,084	0,007	-0,326	0,003	-1,923	0,054
Witkiewitz, 2014	Combined	Blank	0,255	0,266	0,071	-0,266	0,776	0,960	0,337
			0,176	0,138	0,019	-0,094	0,446	1,277	0,202

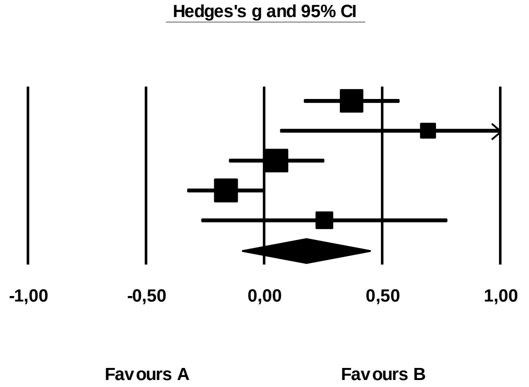

Forest plot of pooled mean effect substance use

Supplementary Figure 9: Forest plot of pooled mean effect self-injury

Study name	Outcome	Comparison	Statistics for each study						
			Hedges's g	Standard error	Variance	Lower limit	Upper limit	Z-Value	p-Value
Franklin, 2016, study 1	suicide ideation	Blank	0,096	0,271	0,074	-0,436	0,628	0,353	0,724
Franklin, 2016, study 2	suicide ideation	Blank	-0,397	0,212	0,045	-0,813	0,019	-1,870	0,062
Franklin, 2016, study 3	suicide ideation	Blank	-0,115	0,224	0,050	-0,554	0,324	-0,513	0,608
Tighe, 2017	DSI-SS	Blank	0,000	0,253	0,064	-0,496	0,496	0,000	1,000
			-0,137	0,118	0,014	-0,369	0,095	-1,159	0,246

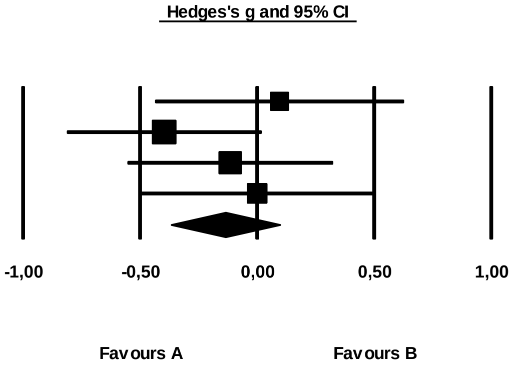

Forest plot of pooled mean effect self-injury

Supplementary Figure 10: Forest plot of pooled mean effect suicidal ideation

Study name	Outcome	Comparison	Statistics for each study						
			Hedges's g	Standard error	Variance	Lower limit	Upper limit	Z-Value	p-Value
Franklin, 2016, study 1	suicide ideation	Blank	0,096	0,271	0,074	-0,436	0,628	0,353	0,724
Franklin, 2016, study 2	suicide ideation	Blank	-0,397	0,212	0,045	-0,813	0,019	-1,870	0,062
Franklin, 2016, study 3	suicide ideation	Blank	-0,115	0,224	0,050	-0,554	0,324	-0,513	0,608
Tighe, 2017	DSI-SS	Blank	0,000	0,253	0,064	-0,496	0,496	0,000	1,000
			-0,137	0,118	0,014	-0,369	0,095	-1,159	0,246

Hedges's g and 95% CI

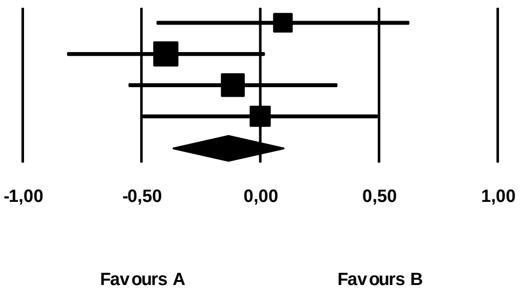

Forest plot of pooled mean effect suicidal ideation

Supplementary Table 1: Risk of bias overview

Trials	Sequence generation	Allocation concealment	Blinding of participants and personnel	Blinding of outcome assessors	Incomplete outcome data	Selective reporting	Other sources of bias
Arean 2016	2	2	0	NA	0	2	Study registration does not match the study<br>High dropout
BinDhim 2016	0	0	0	NA	0	0	
Birney 2016	0	0	1	NA	1	0	
Clarke 2016	1	1	0	NA	1	1	
Dar 2017	2	1	2	NA	1	1	
Enock 2014	2	1	2	NA	1	1	Waitlist came into the study late, study design unclear
Franklin 2016 Study 1	0	1	0	NA	0	1	
Franklin 2016 Study 2	0	1	0	NA	0	1	
Franklin 2016 Study 3	0	1	0	NA	0	1	
Gajecki 2014	0	0	2	NA	2	1	
Horsch 2017	0	0	2	NA	0	0	
Hur 2018	0	1	0	NA	2	1	
Kuhn 2017	0	1	2	NA	0	1	
Miner 2016	1	1	2	NA	0	1	
Pham 2016	0	1	2	NA	1	0	
Roepke 2015	0	0	2	NA	0	1	High dropout
Stolz 2018	0	0	2	NA	0	0	
Tighe 2017	0	0	2	NA	0	0	
Witkiewitz 2014	1	1	2	NA	0	1	

0 = low risk, 1 = unclear risk, 2 = high risk, NA = not applicable

Supplementary Table 2: Outcome overview by study between-group post-assessment

Author year	Outcome [1]	Comparison	Sample size	Hedges' g	95% CI	P
Arean 2016	***Depression***	EVO vs CG	129	0.31	-0.05-0.66	.088
		iPST vs CG	150	0.07	-0.27-.41	.678
BinDhim 2018	***Substance use: smoking***	-	684	0.37	0.17-0.57	≤.001
Birney 2016 [2]	***Depression***	-	199	0.1	-0.18-0.38	.49
Clarke 2016 [3]	***Sleep problems***	-	36	0.84	0.17-1.51	.014
Dar 2017	***Substance use: smoking***	-	40	0.69	.07-1.32	.03
Enock 2014	***Social anxiety***	CBM-A vs CG 1	106	0.65	0.2-1.09	.004
		CBM-A vs CG 2	220	-0.01	-0.28-0.27	.953
	Depression	CBM-A vs CG 1	106	0.48	0.04-0.91	.034
		CBM-A vs CG 2	220	-0.03	-0.3-0.25	.86
Franklin 2016 Study 1	***Self-injury***	-	54	-0.09	-0.62-0.45	.75
	Suicidal ideation	-	54	0.1	-0.44-0.63	.724
Franklin 2016 Study 2	***Self-injury***	-	90	-0.11	-0.52-0.31	.61
	Suicidal ideation	-	90	-0.4	-0.81-0.02	.062
Franklin 2016 Study 3	***Self-injury***	-	81	0.06	-0.38-0.5	.79
	Suicidal ideation	-	81	-0.12	-0.55-0.32	.608
Gajecki 2014	***Substance use: drinking***	PartyPlanner vs CG	398	0.05	-0.15-0.26	.607
		Promillekoll vs CG	586	-0.16	-0.33-0.00	.054
Horsch 2017	***Sleep problems***	-	151	0.72	0.39-1.05	≤.001
	Depression	-	151	0.57	0.25-0.9	≤.05
	Anxiety	-	151	0.65	0.32-0.97	≤.001
Hur 2018	***Depression***	-	34	0.66	-0.01-1.34	.055
	Anxiety	-	34	0.74	0.06-1.42	.034
Kuhn 2017	***PTSD***	-	120	0.14	-0.22-0.5	0.439
	Depression	-	120	0.23	-0.13-0.59	.211
Miner 2016	***PTSD***	-	49	-0.05	-0.6-0.51	.87
Pham 2016	***Anxiety***	-	63	-0.08	-0.57-0.41	.737
Roepke 2015	***Depression***	CBT-PPT SB vs CG	140	0.3	-0.06-0.65	.099
		General SB vs CG	144	0.79	0.43-1.15	≤.001
	Anxiety	CBT-PPT SB vs CG	140	0.28	-0.07-0.64	.113
		General SB vs CG	144	0.64	0.28-0.99	≤.001
Stolz 2018	***Social anxiety***	-	90	0.69	0.19-0.66	.002
	Depression	-	90	0.3	-0.14-0.74	.18
Tighe 2017	***Suicidal ideation***	-	61	0	-0.5-0.5	1
	Depression	-	61	0.71	0.2-1.22	0.007
Witkiewitz 2014	***Substance use: drinking***	-	56	0.23	-0.29-0.75	.392
	Substance use: smoking	-	56	0.34	-0.18-0.86	.204

[1] Primary outcome in **bold**

[2] True sample size between 199-209 discrepancy could not be clarified based on original data

[3] n=36 used in analysis although true sample size was n=35, as unclear in which condition the one missing participant was

Supplementary Methods: Search String Pubmed

Short Description: Search String for PubMed Search sorted by Best Match

Mesh Terms for mobile applications, mental disorders and health, 3 thematic blocks: mhealth, disorders, and study type

Restrictions: Language English or German, Publication Date from January 2007

(mobile applications[MeSH Terms] OR mobile health[Title]OR mhealth[Title]OR m-health[Title]OR app[Title]OR app-based[Title]OR mobile[Title]OR mobile application[Title]OR mobile-based[Title]OR phone-based[Title]OR medical informatics application[Title]OR smartphone[Title]OR smartphone-based[Title] OR pda[Title]OR personal digital assistance[Title]OR tablet[Title])

AND

(mental disorders[MeSH Terms] OR mental health[MeSH Terms] OR mental disorder[Title/Abstract]OR mental disorders[Title/Abstract]OR mental illness* mental disorder[Title/Abstract]OR mental disorders[Title/Abstract]OR mental illness*[Title/Abstract]OR mental health[Title/Abstract]OR psychological health[Title/Abstract]OR CMHD[Title/Abstract]OR mental[Title/Abstract]OR mentally ill[Title/Abstract]OR mental distress[Title/Abstract]OR mental illness[Title/Abstract]OR psychiatric disorder[Title/Abstract]OR psychotic disorders[Title/Abstract]OR affective disorders[Title/Abstract]OR depress*[Title/Abstract]OR anxi*[Title/Abstract]OR anxiety disorder*[Title/Abstract]OR stress*[Title/Abstract]OR stress disorders[Title/Abstract]OR dysthymic disorder[Title/Abstract]OR persistent depressive disorder[Title/Abstract]OR neurotic disorder[Title/Abstract]OR bipolar disorder[Title/Abstract]OR seasonal affective disorder[Title/Abstract]OR mood[Title/Abstract]OR panic[Title/Abstract]OR panic disorder[Title/Abstract]OR agoraphob*[Title/Abstract]OR phobi* [Title/Abstract]OR specific phobi*[Title/Abstract]OR simple phobi*[Title/Abstract]OR phobic[Title/Abstract]OR disorder[Title/Abstract]OR social anxi*[Title/Abstract]OR general* anxi*[Title/Abstract]OR obsess* compuls*[Title/Abstract]OR impulse control disorder*[Title/Abstract]OR somatoform disorders[Title/Abstract]OR somatoform[Title/Abstract]OR sleep disorder*[Title/Abstract]OR sleep disturbance[Title/Abstract]OR sleep problem[Title/Abstract]OR insomnia[Title/Abstract]OR schizophreni*[Title/Abstract]OR psychotic[Title/Abstract]OR psychosis[Title/Abstract]OR psychotrauma[Title/Abstract]OR traumatic[Title/Abstract]OR bipolar [Title/Abstract] OR mania[Title/Abstract]OR self-harm[Title/Abstract]OR suicid*[Title/Abstract]OR addic*[Title/Abstract]OR addiction[Title/Abstract]OR automutilation[Title/Abstract]OR self-injurious behavio*[Title/Abstract]OR smoking cessation[Title/Abstract]OR tobacco use[Title/Abstract]OR gambling[Title/Abstract]OR substance use[Title/Abstract]OR substance-related disorder*[Title/Abstract]OR alcohol-related disorder*[Title/Abstract]OR alcohol abuse alcohol depend*[Title/Abstract]OR alcohol misuse[Title/Abstract]OR medication[Title/Abstract]OR personality attention-deficit[Title/Abstract]OR social[Title/Abstract]OR body[Title/Abstract]OR feeding[Title/Abstract]OR sexual[Title/Abstract]OR disruptive[Title/Abstract]OR cannabis[Title/Abstract]OR eating disorder*[Title/Abstract]OR binge-eating[Title/Abstract]OR anorexia[Title/Abstract]OR bulimia[Title/Abstract]OR bulimi*[Title/Abstract]OR positive psychology[Title/Abstract]OR quality of life[Title/Abstract]OR happiness[Title/Abstract]OR personal satisfaction[Title/Abstract]OR social support[Title/Abstract]OR love[Title/Abstract]OR pleasure[Title/Abstract]OR gratitude[Title/Abstract]OR

compassion[Title/Abstract]OR satisfaction [Title/Abstract]OR life satisfaction[Title/Abstract]OR well-being[Title/Abstract])

AND

(evaluation[Title/Abstract]OR clinical trial[Title/Abstract]OR trial[Title/Abstract]OR randomised control[Title/Abstract]OR randomised controlled[Title/Abstract]OR randomized control[Title/Abstract]OR randomized controlled[Title/Abstract]OR controlled clinical trial[Title/Abstract]OR rando* control*[Title/Abstract]OR controlled[Title/Abstract] OR RCTs[Title/Abstract]OR control protocol[Title/Abstract]OR study protocol[Title/Abstract]OR pilot[Title/Abstract]OR clinical protocol[Title/Abstract]OR RCT[Title/Abstract]OR RCTs[Title/Abstract]OR feasibility[Title/Abstract]OR fesability trial)

Supplementary References: Overview of included studies and study characteristics

1. Arean PA, Hallgren KA, Jordan JT, et al. The Use and Effectiveness of Mobile Apps for Depression : Results From a Fully Remote Clinical Trial Corresponding Author : 18:1-13. doi:10.2196/jmir.6482.
2. BinDhim NF, McGeechan K, Trevena L. Smartphone Smoking Cessation Application (SSC App) trial: a multicountry double-blind automated randomised controlled trial of a smoking cessation decision-aid "app". *BMJ Open*. 2018;8(1):e017105. doi:10.1136/bmjopen-2017-017105.
3. Birney AJ, Gunn R, Russell JK, Ary D V. MoodHacker Mobile Web App With Email for Adults to Self-Manage Mild-to-Moderate Depression: Randomized Controlled Trial. *JMIR mHealth uHealth*. 2016;4(1):e8. doi:10.2196/mhealth.4231.
4. Clarke P, Bedford K, Notebaert L, et al. Assessing the Therapeutic Potential of Targeted Attentional Bias Modification for Insomnia Using Smartphone Delivery. *Psychother Psychosom*. 2016;85(3):187-189. doi:10.1159/000442025.
5. Dar R. Effect of Real-Time Monitoring and Notification of Smoking Episodes on Smoking Reduction: A Pilot Study of a Novel Smoking Cessation App. *Nicotine Tob Res*. 2017;(March):1-4. doi:10.1093/ntr/ntx223.
6. Enock P, Hofmann S, McNally R. Attention bias modification training via smartphone to reduce social anxiety: a randomized, controlled multi-session experiment. *Cognit Ther Res*. 2014;38(2):200-216. doi:10.1007/s10608-014-9606-z.
7. Franklin JC, Fox KR, Franklin CR, et al. A brief mobile app reduces nonsuicidal and suicidal self-injury: Evidence from three randomized controlled trials. *J Consult Clin Psychol*. 2016;84(6):544-557. doi:http://dx.doi.org/10.1037/ccp0000093.
8. Gajecki M, Berman AH, Sinadinovic K, Rosendahl I, Andersson C. Mobile phone brief intervention applications for risky alcohol use among university students: a randomized controlled study. *Addict Sci Clin Pract*. 2014;9:11. doi:10.1186/1940-0640-9-11.
9. Horsch CH, Lancee J, Griffioen-Both F, et al. Mobile Phone-Delivered Cognitive Behavioral Therapy for Insomnia: A Randomized Waitlist Controlled Trial. *J Med Internet Res*. 2017;19(4):e70. doi:10.2196/jmir.6524.
10. Hur J-W, Kim B, Park D, Choi S-W. A Scenario-Based Cognitive Behavioral Therapy Mobile App to Reduce Dysfunctional Beliefs in Individuals with Depression: A Randomized Controlled Trial. *Telemed e-Health*. 2018;24(10):tmj.2017.0214. doi:10.1089/tmj.2017.0214.
11. Kuhn E, Kanuri N, Hoffman JE, Garvert DW, Ruzek JI, Taylor CB. A randomized controlled trial of a smartphone app for posttraumatic stress disorder symptoms. *J Consult Clin Psychol*. 2017;85(3):267-273. doi:10.1037/ccp0000163.
12. Miner A, Kuhn E, Hoffman JE, Owen JE, Ruzek JI, Taylor CB. Feasibility, acceptability, and potential efficacy of the PTSD Coach app: A pilot randomized controlled trial with community trauma survivors. *Psychol Trauma*. 2016;8(3):384-392. doi:10.1037/tra0000092.
13. Pham Q, Khatib Y, Stansfeld S, Fox S, Green T. Feasibility and Efficacy of an mHealth Game for Managing Anxiety: "Flowy" Randomized Controlled Pilot Trial and Design Evaluation. *Games Health J*. 2016;5(1):50-67. doi:10.1089/g4h.2015.0033.
14. Roepke AM, Jaffee SR, Riffle OM, McGonigal J, Broome R, Maxwell B. Randomized Controlled Trial of SuperBetter, a Smartphone-Based/Internet-Based Self-Help Tool to Reduce Depressive Symptoms. *Games Health J*. 2015;4(3):235-246. doi:10.1089/g4h.2014.0046.
15. Stolz T, Schulz A, Krieger T, et al. A mobile App for social anxiety disorder: A three-arm randomized controlled trial comparing mobile and PC-based guided self-help interventions. *J Consult Clin Psychol*. 2018;86(6):493-504. doi:10.1037/ccp0000301.
16. Tighe J, Shand F, Ridani R, MacKinnon A, De La Mata N, Christensen H. Ibobbly mobile health intervention for suicide prevention in Australian Indigenous youth: A pilot randomised controlled trial. *BMJ Open*. 2017;7(1). doi:10.1136/bmjopen-2016-013518.
17. Witkiewitz K, Desai SA, Bowen S, Leigh BC, Kirouac M, Larimer ME. Development and evaluation of a mobile intervention for heavy drinking and smoking among college students. *Psychol Addict Behav*. 2014;28(3):639-650. doi:10.1037/a0034747.
